# Individualized Housing Modifies the Immune–Endocrine System in CD1 Adult Male Mice

**DOI:** 10.3390/ani13061026

**Published:** 2023-03-10

**Authors:** Iván Ortega-Saez, Alina Díez-Solinska, Roger Grífols, Cristina Martí, Carolina Zamora, Maider Muñoz-Culla, Oscar Vegas, Garikoitz Azkona

**Affiliations:** 1 Parc de Recerca Biomèdica de Barcelona (PRBB) Animal Facility, 08003 Barcelona, Spain; 2 Department of Basic Psychological Processes and Their Development, Euskal Herriko Unibertsitatea (UPV/EHU), 20018 Donostia, Spain; 3 Charles River Laboratories, Parc de Recerca Biomèdica de Barcelona (PRBB), 08003 Barcelona, Spain; 4 Biodonostia Health Research Institute, 20014 Donostia, Spain

**Keywords:** CD1 male, single-housed, stress, white blood cells, fecal corticosterone metabolites

## Abstract

**Simple Summary:**

In recent years, awareness of laboratory animals’ wellbeing and the refinement of their house conditions have increased considerably. Mice (*Mus musculus*) are the most widely used animal species in research in the European Union and are sociable and hierarchical creatures. It is important to determine whether experimental conditions may affect research results and whether housing conditions (isolated or grouped) may be one such condition. The aim of this study was, therefore, to determine whether 4 weeks of social isolation (usual practice in our animal facility and some laboratory procedures) could induce changes in different physiological parameters (body weight, number of blood cells, and stress hormones) in adult mice. Although we did not observe changes in body weight, red blood cells, and platelets, mice that were socially isolated for 4 weeks did have a decreased count of some white blood cells. Moreover, levels of the main stress hormone were higher in single-housed mice after 1 week, although they decreased after 4 weeks to the same levels as those recorded for grouped mice. We can, therefore, conclude that social isolation affects some physiological parameters, and that this should be taken into account in the interpretation of research data.

**Abstract:**

In the last years, different research groups have made considerable efforts to improve the care and use of animals in research. Mice (*Mus musculus*) are the most widely used animal species in research in the European Union and are sociable and hierarchical creatures. During experiments, researchers tend to individualize males, but no consideration is given to whether this social isolation causes them stress. The aim of this study was, therefore, to explore whether 4 weeks of social isolation could induce changes in different physiological parameters in adult Crl:CD1(ICR) (CD1) males, which may interfere with experimental results. Body weight, blood cells, and fecal corticosterone metabolites levels were the analyzed parameters. Blood and fecal samples were collected at weeks 1 and 4 of the experimental procedure. Four weeks of single housing produced a significant time-dependent decrease in monocytes and granulocytes. Fecal corticosterone metabolite levels were higher in single-housed mice after 1 week and then normalized after 4 weeks of isolation. Body weight, red blood cells, and platelets remained unchanged in both groups during this period. We can, therefore, conclude that social isolation affects some immune and endocrine parameters, and that this should be taken into account in the interpretation of research data.

## 1. Introduction

People working with laboratory animals display a high level of awareness of and sensitivity to their wellbeing [1]. Indeed, perceived animal stress/pain has been found to negatively affect their professional quality of life [2]. In the last few years, different research groups have made considerable efforts to improve the care and use of animals in research, regardless of receiving specific funding for that purpose [3]. In the near future, this new scientific knowledge will provide new evidence to improve the welfare and housing conditions of animals used in scientific procedures. Current European legislation on the protection of animals used for scientific purposes (Directive 210/63/EU) establishes suitable environmental conditions and minimum enclosure measures by age and animal species. It likewise indicates that social laboratory animals must be socially housed in stable groups of compatible individuals. Moreover, procedures in which social animals (e.g., dogs and monkeys) are completely isolated for prolonged periods are classified as “severe” [4]. However, the legislation does not specify what exactly is considered to be a “prolonged period”, and it does not mention other social species.

Despite the current debate about their predictive value in basic and regulatory studies [5,6,7,8,9,10], mice (*Mus musculus*) continue to be the most widely used animal species in research in the European Union [11]. Mice are sociable and hierarchical animals that, in nature, live in small groups. These groups are usually composed of a dominant male, along with various females with their offspring, both young and juvenile. The size of the territory occupied by a mouse family varies according to different factors. These include the availability of different resources such as water and food, as well as the density of the group. Occasionally, depending on the aggressiveness of the dominant male and the density of the group, young males are found in the aforementioned family groups. Generally, however, males are usually rejected from the group when they reach sexual maturity and can be found in the wild alone or in groups of young males. As for the females, they usually become part of the family group once they reach sexual maturity [12].

Unfortunately, in animal facilities, mice are not housed as in their natural environment, thus interfering with their natural ethogram. Standard laboratory protocols stipulate that mice’s weaning and maternal separation should occur 21 days after birth. Thereafter, it is recommended that animals should be housed separately by sex and strain in stable groups of 2–5 members, a step that fosters the formation of affiliate relationships between individuals in the same group [13] and reduces aggression between males [14]. The main reason for housing male mice individually is aggression between cage mates [15,16]. Recently, a series of recommendations were published to minimize aggression between males [17].

Keeping newly weaned animals in the company of other animals is important for the correct development of their brains. It has been shown that post-weaning social deprivation by isolating mice induces neurochemical and morphological alterations, which have a behavioral impact in adulthood [13,18,19,20,21,22,23]. Indeed, the lack of social experiences before adulthood has been used in mice as a model to study some impaired behavioral phenotypes, such as depression and anxiety-like behavior types [21,22,23], as well as social and cognitive deficits [19,22]. In light of the above, in our animal facility, we implemented two different strategies in order to minimize the number of single-housed newly weaned male mice [24,25].

There is still an ongoing debate about whether adult male mice should be housed individually [15,26]. Years ago, “isolation syndrome” was described, with authors arguing that the inability to interact socially is likely to have a harmful effect on the animal’s emotional state [27]. Indeed, it has been proven that adult male mice prefer the proximity of another male over individual housing [28], which is considered a stressor. The gold standard to measure the immediate physiological responses to stress is the activation of the hypothalamic–pituitary–adrenal (HPA) axis, which induces the secretion of corticosterone from the adrenal gland [29]. The effect of solitary versus social housing on corticosterone levels has been explored with varying results. Some studies observed that single-housed male mice had increased corticosterone levels after 14 days [30] and 15 months [31], whereas others found that corticosterone levels remained stable up to 42 days of individual housing [32,33,34,35,36], and two studies reported that single housing caused less stress for mice than group housing [37,38]. Other indications of stress include changes in body weight and a decrease in circulating leukocytes. A meta-analysis of the effects of individual housing on body weight found considerable heterogeneity in different mice strains, with higher, unchanged, or lower body weights being reported after social isolation [39]. Although it is well documented that chronic stress results in immunosuppression [40], differences in the total number of white blood cells have also been observed [36,41]. Among other factors, these discrepancies may be due to differing isolation periods.

In our animal facility, researchers tend to individualize males during experiments for a maximum period of 4 weeks, mainly for reasons of convenience and habit. However, no consideration is given to whether individually housing animals may cause them stress. The aim of the present study was, therefore, to determine if 4 weeks of social isolation could induce changes in body weight, blood cells, or fecal corticosterone metabolite levels in adult Crl:CD1(ICR) (CD1) males, which may interfere with experimental results.

## 2. Materials and Methods

### 2.1. Animals

Mice born in our specific pathogen-free (SPF) breeding zone were housed in pressurized and individually ventilated 1145T (403 × 165 × 174 mm; 435 cm^2^ floor area; Tecniplast) (PIV) cages (70 air changes/h). We used black poplar/aspen shavings (Lignocel Selectfine; Rettenmaier Ibérica S.L.) as litter bedding, two sheets of tissue (Tork^®^; Essity Spain S.L) irradiated by Ionisos Iberica as nesting material, and an in-house autoclaved cardboard cylinder (12.5 × 9 × 0.5 cm; Sodispan Research S.L.) as enrichment. Once a week, socially housed mice (four mice per cage), together with their nesting material, were transferred to clean cages by picking them up at the base of their tails. This same procedure was carried out with individually housed mice every other week. New irradiated tissue was added if the nest was dirty or did not have enough material. Similarly, if the cardboard was broken, a new cylinder was provided. Mice had ad libitum access to water and diet (irradiated Special Diet Services RM1). Rooms were maintained under standard environmental conditions (humidity: 55 ± 10%; temperature: 20–24 °C) with a 12 h light/dark cycle (lights on at 8:00 a.m.). Animals were monitored every day. The animal care and use program was accredited by AAALAC International. The Catalan Government and the PRBB Ethics Committees approved the experimental protocol (DAAM 10576).

### 2.2. General Procedure

Eight-week-old CD1 mice were randomly assigned to two groups (grouped or single; n = 8 per group, 16 in total) and housed in the same room in which they were born. We selected CD1 adult male mice because they are outbred, are the most commonly used strain in toxicology studies [42], and have a high propensity to fight, resulting in suggestions that they may benefit from individual housing [15]. This does not apply to females, since chronic social isolation is used to model separation-induced depression [43].

**Figure 1:**
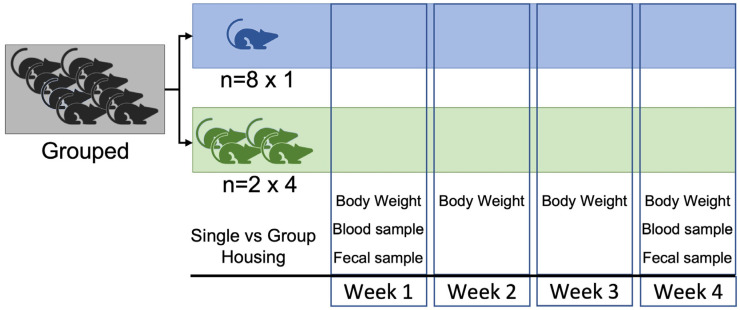
Experimental procedure.

Animals were weighed on the same day of the week for 5 weeks (weeks 0–4; 9:00–11:00 a.m.). Sampling was carried out in a laboratory adjacent to the room where they were housed, and the animals were transferred there 1 h before sampling, around 8:00 a.m., because the technician started their working day at this time. Sampling was carried out at two different time points to minimize the influence of handling as much as possible. Thus, on weeks 1 and 4 (9:00–11:00 a.m.), whole blood and fecal samples were obtained from each animal (Figure 1). No signs of fighting were observed during the experimental period. None of the animals had adverse events, and all completed the procedure. Animals became part of our colony once the experiment was completed.

### 2.3. Hematological Parameters

Blood samples were obtained by facial vein puncture with a 21 G sterile hypodermic needle. We collected blood from the facial vein because this procedure has been found to have the least adverse effects on welfare parameters in mice [44,45]. Samples (15 μL) were collected using a Microvette^®^ 200K3E with potassium salt of ethylenediaminetetraacetic acid (EDTA) as an anticoagulant. After sampling, mice were returned to their home cage. No residual bleeding was noted in any of the animals. The blood was immediately analyzed for complete blood count: white blood cells (WBC), lymphocytes, monocytes, granulocytes, red blood cells (RBC), hemoglobin (HGB), hematocrit (HCT), mean corpuscular volume (MCV), hemoglobin (MCH) and hemoglobin concentration (MCHC), red cell distribution width (RDW), platelets (PLT), mean platelet volume (MPV), platelet distribution width (PDW), and platelet crit (PCT), using the fully automated CVM-Procell analyzer (CVM Diagnóstico Veterinario SL). Since the provider could not give us information about the exact mouse strain, age, or sex where the values were obtained, we first determined if the blood value range of male and female adult mice of different commonly used strains were within the normal range indicated by the analyzer. Our results indicated that the normal range provided for mice by the CVM-Procell analyzer can be used for adult male and female inbred C57BL/6J, outbred CD1, and immunodeficient CB17.Cg-*Prkdc^scid^Lyst^bg-J^*/Crl (SCID Beige) mice (see Supplementary Materials).

### 2.4. Fecal Corticosterone Metabolites

Fecal samples were obtained by placing each animal on a grid. The fecal boluses were obtained directly, without possible contamination, placed in an Eppendorf, and stored at −80 °C to determine corticosterone metabolite levels. After sampling, mice were returned to their home cage. This sampling method may allow a more accurate interpretation of chronic stress [46]. Moreover, since there is no need to restrain the animals when collecting the samples, this is a good method for enabling repeated sampling without affecting the animal, meaning that fecal samples are less affected by hormone secretion fluctuation or pulsatility. Each fecal sample was homogenized, and an aliquot of 0.05 g was shaken with 1 mL of 80% methanol in Tris/HCl 20 mM, pH 7.5, for 30 min on a multi-vortex. After centrifugation, each aliquot was frozen at −80 °C until analysis. Fecal corticosterone metabolite levels were quantified in duplicate using an enzyme immunoassay (Corticosterone Elisa Kit, Enzo Life Sciences; ADI-900-097), in accordance with the manufacturer’s recommendations, and a Synergy HT microplate reader (BioTek Instruments, Inc., Winooski, VT, USA). Data were analyzed by means of a four-parameter logistic curve fit using MyAssays (Data Analysis Tools and Services for Bioassays; available at https://www.myassays.com/ accessed on 10 March 2023). The sensitivity of the assay was 27.0 pg/mL, and the intra- and inter-assay variation coefficients were between 7% and 8%.

### 2.5. Statistical Analyses

Experimental data were analyzed using GraphPad Prism software (6.01, GraphPad Software, Inc, San Diego, CA, USA). Group comparisons were performed using a two-way repeated-measures ANOVA, followed by Bonferroni’s post hoc test. Values of *p* < 0.05 were considered statistically significant (95% confidence). Data are expressed as the mean ± standard deviation (SD). The results are described in accordance with the ARRIVE guidelines [47].

## 3. Results

### 3.1. Body Weight

**Figure 2:**
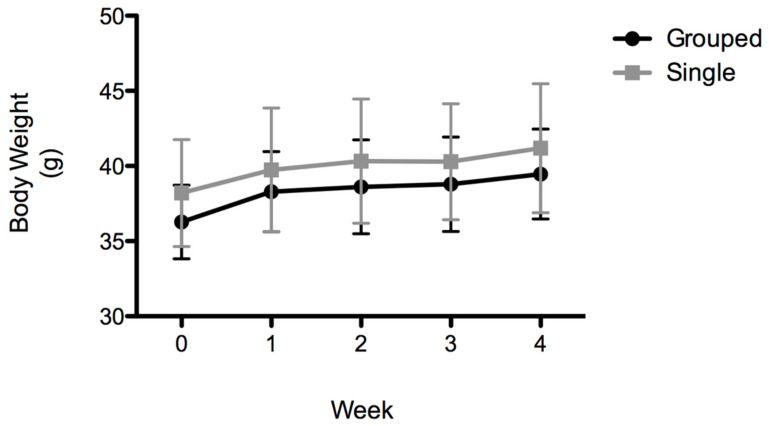
Body weight (g). Data are expressed as the mean ± SD; n = 8 per group.

Both groups of animals gained weight over the duration of the experiment (F_(4,56)_ = 34,78, *p* < 0.0001). Grouped mice weighed 36.27 ± 2.46 g at week 0 and 39.46 ± 2.99 g at week 4. Single-housed mice weighed 38.20 ± 3.55 g at week 0 and 41.19 ± 4.29 g at week 4 (Figure 2). No significant differences were observed between grouped or single-housed mice.

### 3.2. Hematological Parameters

The results indicated no significant differences between grouped and single mice in the number of cells in the white series at either week 1 or week 4. However, significant differences were observed as a function of time (F_(1,14)_ = 5.52; *p* < 0.05; Table 1). The post hoc analysis indicated a significant decrease in WBC after 4 weeks of single housing (t = 2.21; *p* < 0.05). When white cell type was analyzed in more detail, significant time-dependent differences were observed in monocytes (F_(1,14)_ = 10.45; *p* < 0.01), and the post hoc analysis indicated a significant drop in monocytes in single-housed mice after 4 weeks (t = 2.714 *p* < 0.05). Similarly, significant time-dependent differences were observed in granulocytes (F_(1,14)_ = 7.63; *p* < 0.05), which dropped in single-housed mice after 4 weeks (t = 2.46, *p* < 0.05).

The results indicated no significant differences between groups or timepoints in terms of the number of red blood cells and platelets (Table 2).

### 3.3. Fecal Corticosterone Metabolites

**Figure 3:**
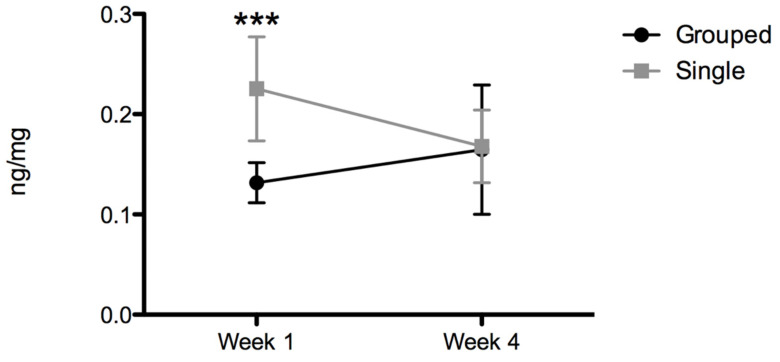
Fecal corticosterone metabolites (ng/mg feces). Data are expressed as the mean ± SD; n = 8 per group; *** *p* < 0.001.

The statistical study of fecal corticosterone metabolite levels revealed a significant interaction between variables (F_(1,14)_ = 11,40, *p* < 0.01). The post hoc analysis indicated significantly higher corticosterone metabolite levels in single-housed (0.225 ± 0.05 ng/mg) than in grouped animals (0.132 ± 0.02 ng/mg) after 1 week (t = 4.523; *p* < 0.001). At 4 weeks, no differences were observed between groups (grouped: 0.165 ± 0.06 ng/mg vs. single: 0.168 ± 0.04 ng/mg; t = 0.488, *p* > 0.05), and single-housed corticosterone metabolite levels were normalized (Figure 3).

## 4. Discussion

It is well known that animal welfare has an effect on the outcome of experiments. We must, therefore, always consider this factor when designing and carrying out experimental procedures. However, many researchers systematically tend to individualize animals in their experiments. Thus, the question we aimed to answer in this study was whether a lack of social interaction may modify physiological parameters, which may in turn interfere with experimental results. Our findings indicate that social isolation modifies some physiological parameters.

As previously reported for CD1 male mice [48,49,50], social isolation for 4 weeks did not affect body weight gain. Similarly, our results revealed that social isolation did not modify RBC parameters. As far as we are aware, this is the first study in mice to analyze RBC parameters; thus, we cannot compare our results with previous findings.

Mice that were changed from sharing a cage with littermates to living alone showed higher fecal corticosterone metabolites than those maintained in the group after the first week, although levels normalized after 1 month. These same results were recently observed in adult CD1 mice housed in the same conditions as our animals, in a ventilated rack with environmental enrichment [50], which may indicate habituation to the new situation. Due to the nature of our experimental design, we were unable to determine when exactly corticosterone metabolite levels normalized, and this is one of our study’s limitations. However, data from a previous study [33] indicated that fecal corticosterone metabolite levels start to decrease and remain stable from the second week onward. These data are consistent with those described previously in relation to the return of plasma glucocorticoids to baseline values during the first week after transport or translocation [51,52,53,54]. Among the grouped animals, no significant changes were observed across individuals, and the standard deviation within groups was very small. Our data, therefore, seem to suggest that, in contrast to observations by some authors [37,38], remaining grouped together does not appear to cause the animals any stress. We believe the main reason for this is that, as has indeed been pointed out previously [32], our mice were littermates and were grouped together from weaning.

It is well known that increased glucocorticoid levels suppress cellular immunity [55]. No changes in monocytes and granulocytes were observed in single-housed animals after 7 days, although changes were found after 4 weeks. A previous study found no significant differences in the overall number of blood-circulating leukocytes between CD1 male mice that were socially isolated for 2 weeks and their socially housed counterparts [36]. However, C57BL6/J adult mice separated into individual cages for 2 h every day for 25 days were found to have a decrease in T cells, B cells, monocytes, and neutrophils [41]. Unfortunately, our system is not able to distinguish between the different types of lymphocytes and granulocytes; however, overall, our results are consistent with these findings and highlight the fact that isolation time is a factor to be considered. Another limitation of the study is that we did not study humoral immunity; previous studies found that fecal immunoglobulin A (IgA) excretion (a marker of long-term stress) takes at least 4 weeks to normalize [53]. It is important to note that CD1 adult males isolated for 21 days and subjected to mild psychological stress had lower splenocyte proliferation and lower IL-2 and IL-4 cytokine plasma levels than their grouped counterparts [32]. The same results were reported using shock as a stressor [55].

In addition to the limitations outlined above, our study had some further limitations. When designing the experiment, we wanted it to be as realistic as possible in terms of the day-to-day management of our animal facility technicians and researchers. Therefore, the animals were moved from dirty to clean cages by picking them up by the tail. In recent years, less aversive handling methods (e.g., tunnel or cup handling) have been shown to mitigate anxiety and depressive-like behaviors [56,57,58]. However, a recent study showed that picking mice up by their tail may not be a significant source of chronic husbandry stress [59]. In view of the results of this study and our daily practice, we decided to change the location of animals in this experiment by picking them up by their tail. We are all aware that efforts have to be made to implement less aversive methods of handling in daily practice in animal facilities. Nevertheless, it should also be kept in mind that this procedure takes more time; hence, the amount of work assigned to each technician when changing cages should also be reviewed. In our work, we did not study whether social isolation induced behavioral changes in our animals, because we were more interested in peripheral biomarkers than behavioral parameters. In a recent study performed on C57BL/6JRj mice housed singly for 10 weeks, no behavioral changes were observed in exploratory activity, anxiety, working memory, and fear memory [60]. However, a previous study using C57BL/6J and DBA/2 kept in individual housing for 7 weeks revealed that individual housing has strong strain- and test-specific effects on emotional behavior and impaired memory in certain tasks. Single-housed mice were hyperactive and displayed reduced habituation to novel environments. Reduced anxiety was established in the elevated plus-maze, but not in the dark/light test. Immobility in the forced swimming test was reduced by social isolation. Novel object recognition and fear conditioning were impaired in the single-housed mice, whereas water-maze learning was not affected [61]. In the same way, 2 weeks of single housing plus acute injection stress induced anxiety-like behavior in C57BL6/J mice [30]. Mouse strain and social environment also influence depression-like behavior caused by an immune challenge. In this sense, group-housed CD1 mice exhibited depression-like behavior 1 day after bacterial lipopolysaccharide (LPS) injection, while the behavior of single-housed CD1 mice was little affected during the 4 weeks of the experiment. In contrast, both grouped and single-housed C57BL/6 mice responded to LPS with an increase in depression-like behavior [62]. It would be interesting to conduct future behavioral studies to determine if, under our conditions, single-housed CD1 male mice show any behavioral changes. Another parameter we did not measure was body temperature. In recent years, it has been observed that laboratory mice suffer from thermal stress, and that this affects their immune system, among other physiological parameters [63,64]. In this sense, huddling, a form of social thermoregulation, is a major contributor to mice’s thermal physiology. Thus, single-housed mice are usually more affected by cold temperatures than grouped mice [65]. In order to mitigate this effect, two sheets of tissue were added to their home cage, and we ensured that they made a proper nest.

In light of all these data, we recommend keeping males in stable groups from weaning onward. Researchers should be aware that the change from grouping to living alone induces stress and mild immunosuppression in CD1 male mice; hence, if the mice need to be separated for experimental reasons, these factors should be taken into consideration.

## 5. Conclusions

We conclude that social isolation has an effect on the immune–endocrine system. Consequently, the stress associated with the new social situation should be taken into consideration in the interpretation of research data.

## Figures and Tables

**Table 1:** White blood cell population values. Data are expressed as the mean ± SD; n = 8 per group; * *p* < 0.05 (week 1 single vs. week 4 single).

	Week 1		Week 4		Normal Range	Unit
	Grouped	Single	Grouped	Single		
WBC	8.45 ± 3.33	8.15 ± 4.59	7.18 ± 2.59	4.50 ± 1.83 *	0.8–6.8	10^9^/L
Lymph	5.81 ± 2.42	5.73 ± 3.08	4.76 ± 0.81	4.13 ± 0.52	0.7–5.7	10^9^/L
Mon	0.33 ± 0.14	0.34 ± 0.31	0.26 ± 0.11	0.10 ± 0.08 *	0.0–0.3	10^9^/L
Gran	2.33 ± 0.93	1.96 ± 1.50	1.70 ± 0.48	1.07 ± 0.56 *	0.1–1.8	10^9^/L

* White blood cell (WBC), lymphocyte (Lymph), monocyte (Mon), and granulocyte (Gran).

**Table 2:** Red blood cell and platelet values.

	Week 1		Week 4		Normal Range	Unit
	Grouped	Single	Grouped	Single		
RBC	8.79 ± 1.18	8.32 ± 1.42	8.76 ± 0.95	8.53 ± 0.93	6.36–9.42	10^12^/L
HGB	14.59 ± 1.85	13.81 ± 2.79	14.73 ± 1.67	13.71 ± 1.42	11–14.3	g/dL
HCT	43.20 ± 5.24	42.15 ± 6.79	44.08 ± 4.64	42.09 ± 4.65	34.6–44.6	%
MCV	49.30 ± 1.09	50.83 ± 0.98	50.20 ± 1.69	49.41 ± 1.88	48.2–58.3	fL
MCH	16.58 ± 1.42	16.38 ± 0.42	16.46 ± 0.36	14.79 ± 0.78	15.8–19	pg
MCHC	337.13 ± 4.78	325.63 ± 5.06	328.86 ± 18.8	325.63 ± 11.4	302–353	g/L
RDW	13.30 ± 0.77	14.74 ± 0.33	13.55 ± 1.13	12.86 ± 1.27	13–17	%
PLT	1021.8 ± 582.3	762.4 ± 498.0	1140.0 ± 226.6	892.4 ± 273.7	450–1590	10^9^/L
MPV	4.91 ± 0.35	5.39 ± 0.15	5.31 ± 0.430	5.13 ± 0.53	3.8–6	fL
PDW	16.88 ± 1.10	17.31 ± 1.47	16.67 ± 1.14	17.21 ± 1.28	-	-
PCT	0.41 ± 0.20	0.30 ± 0.27	0.44 ± 0.25	0.31 ± 0.23	-	%

Hemoglobin (HGB), hematocrit (HCT), mean corpuscular volume (MCV), hemoglobin (MCH) and hemoglobin concentration (MCHC), red cell distribution width (RDW), platelets (PLT), mean platelet volume (MPV), platelet distribution width (PDW), and platelet crit (PCT).

## Data Availability

All the data of the study can be made available upon request.

## Supplementary Materials

The following supporting information can be downloaded at https://www.mdpi.com/article/10.3390/ani13061026/s1: Determination of CVM-Procell analyzer reference values for each strain. References [66,67,68,69,70,71] are cited in the supplementary materials
