# Supplementary material for: Individualized Housing Modifies the Immune–Endocrine System in CD1 Adult Male Mice
Source: Animals (Basel). 2023 Mar 10;13(6):1026. doi: 10.3390/ani13061026 (PMC10044133; doi:10.3390/ani13061026)
Supplement: Supplementary file 1 [file animals-13-01026-s001.zip › animals-2267239-supplementary.pdf]

Determination of CVM-Procell analyzer reference values for each strain

Aim of the study

The provider of the CVM-Procell analyzer could not give us information about the exact mouse strain, age, or sex where the values were obtained. Thus, we determine the blood values of adult males and females (9-12 weeks-old) of inbred C57BL/6J, outbred Crl:CD1(ICR) (CD1), and immunodeficient CB17.Cg-*Prkdc*^{scid}*Lyst*$^{bg\text{-}J}$/Crl (SCID Beige) were in the normal range the analyzer indicates for mice.

Material and Methods

Animals

Mice born in our specific pathogen-free (SPF) breeding zone were housed in 1145T (Tecniplast) individually ventilated cages, with poplar/aspen shavings as litter, irradiated tissues as nesting material and autoclaved cardboard cylinders as enrichment. Once a week, mice, together with their nesting material, were transferred to clean cages. Mice had *ad libitum* access to water and diet (irradiated Special Diet Services RM1). Rooms were maintained under standard environmental conditions (humidity: 40-70%; temperature: 20-24ºC) and a 12 h light/dark cycle (lights on at 8:00 a.m.). Animals were monitored every day. The animal care and use program is accredited by AAALAC International. The Catalan Government and PRBB Ethics Committees approved the experimental protocol (DAAM 10576).

General procedure

We got samples from 120 animals, 20 male and 20 female mice of each strain. Whole blood samples were obtained by facial vein puncture with a 21G sterile hypodermic needle. Samples (15 µL) were collected using a Microvette® 200K3E with potassium salt of ethylene dimetilenotetracetic acid (EDTA) as an anticoagulant. After sampling, mice were returned to their home cage. No residual bleeding was noted in any of the animals. The blood was immediately analyzed for complete blood count; white blood cell (WBC), lymphocytes, monocytes, granulocytes, red blood cell (RBC), hemoglobin (HGB), hematocrit (HCT), mean corpuscular volume (MCV), hemoglobin (MCH) and hemoglobin concentration (MCHC), red cell distribution width (RDW), platelets (PLT), mean platelet volume (MPV), platelet distribution width (PDW) and platelet crit (PCT) using the fully automated CVM-Procell analyzer (CVM Diagnóstico Veterinario SL, Tudela, Spain).

Results and Discussion

White blood cell (WBC) count consisted of the total and relative number of different leukocyte populations; lymphocytes, monocytes, and granulocytes (Table 1). The results showed that there were no sex differences in any strain. As expected, immunodeficient mice due to their mutations SCID (Prkdcscid) and beige (Lystbg) [66] showed significantly lower WBC ($F_{(2,114)}$ = 74.20), lymphocytes ($F_{(2,114)}$ = 114.7), and monocytes ($F_{(2,114)}$ = 21.71) compared

to immunocompetent strains ($p < 0.0001$). Besides, lymphocyte number and percentage in immunodeficient mice were lower than the normal range, whereas granulocyte percentage was higher in both sexes. C57BL/6J and CD1 mice leukocyte values were within the normal range.

Table S1. White blood cell values. Mean ± SD and CVM-Procell analyzer normal range of C57BL/6J, CD1, and SCID Beige male and female adult mice, from 9 to 12 weeks of age (n = 20 per group).

	C57BL/6J		CD1		SCID BEIGE		Nornal Range	Unit
	Male	Female	Male	Female	Male	Female		
WBC	5.85±2.25	5.05±1.63	6.82±2.04	5.60±2.63	1.80±0.09***	1.47±0.14***	0.8-6.8	10^9/L
Lymph	4.47±1.95	3.83±1.27	4.92±1.52	4.16±1.74	0.41±0.14***	0.41±0.14***	0.7-5.7	10^9/L
Mon	0.20±0.11	0.18±0.07	0.23±0.10	0.15±0.11	0.09±0.05***	0.05±0.05***	0.0-0.3	10^9/L
Gran	1.64±0.80	1.33±0.35	1.68±0.61	1.39±0.92	1.58±0.38	0.99±0.47	0.1-1.8	10^9/L

***Statistically significant lower values compared to C57BL/6J and CD1 mice ($p < 0.0001$).

Concerning the red blood cell line, SCID Beige mice values were statistically different compared with the other strains (Table 2). Immunodeficient mice showed higher RBC ($F_{(2,114)} = 6.768$; $p < 0.001$), HGB ($F_{(2,114)} = 8.086$; $p < 0.0001$) and HCT ($F_{(2,114)} = 3.577$; $p < 0.05$), but lower MCV ($F_{(2,114)} = 56.38$; $p < 0.0001$) and RDW ($F_{(2,114)} = 56.80$; $p < 0.0001$). Thus, the levels were a little above (for counts and hemoglobin content) or below (for volume) the normal range provided by the analyzer, so the anomaly does not fulfill the criteria for either polycythemia or anemia. Hematological values of immunocompetent mice were within the normal range, as reported using other automated analyzers [67, 68, 69]. However, our results did not show previously reported significant differences between C57BL/6J male and female mice [70]. This could be due to the difference in the sample site collection [71], retro-orbital sinus versus facial vein puncture, or in the analyzer, ABX Pentra 60C+ Analyser (Horiba ABX, Montpellier, France) versus CVM-Procell. Regarding the sampling method, we collected the blood from the facial vein because it has been reported that it had the least adverse effects on welfare parameters in mice [44, 45].

Table S2. Red blood cells and platelet values. Mean ± SD and CVM-Procell analyzer normal range of C57BL/6J, CD1, and SCID Beige male and female adult mice, from 9 to 12 weeks of age (n = 20 per group).

	C57BL/6J		CD1		SCID BEIGE		Nornal Range	Unit
	Male	Female	Male	Female	Male	Female		
RBC	8.69±1.28	8.90±0.67	8.75±1.40	8.77±0.80	9.43±0.52**	9.43±0.30**	6.36-9.42	10^12/L
HGB	13.57±2.20	14.16±2.41	14.22±2.66	14.08±1.82	15.71±1.63***	15.62±0.44***	11-14.3	g/dL
HCT	42.04±6.63	43.99±3.28	44.05±4.48	44.01±3.94	45.37±4.16*	45.49±0.95*	34.6-44.6	%
MCV	49.07±1.09	48.91±0.98	49.84±1.69	50.28±1.88	47.37±1.06***	47.54±0.69***	48.2-58.3	fL

MCH	16.69±1.42	16.69±0.42	16.53±0.36	16.98±0.78	16.32±0.69	16.26±0.35	15.8-19	pg
MCHC	337.35±4.78	341±5.06	336.65±18.8	337.45±11.4	345.45±8.50	342±5.52	302-353	g/L
RDW	14.82±0.77	15.01±0.33	14.13±1.13	13.38±1.27	12.98±0.52***	12.94±0.54***	13-17	%
PLT	949.0±211.6	896.1±157.8	1034.3±445.6	954.5±449.7	1122.8±147.6	958.0±96.8	450-1590	10^9/L
MPV	5.12±0.35	4.81±0.15	5.03±0.43	5.01±0.53	5.23±0.43	4.93±0.28	3.8-6	fL
PDW	16.57±1.10	15.93±0.14	16.69±1.14	16.74±1.28	16.48±0.49	16.48±0.35	-	-
PCT	0.33±0.20	0.42±0.07	0.36±0.25	0.29±0.23	0.47±0.18	0.44±0.10	-	%

Statistically significant higher levels or lower values compared to C57BL/6J and CD1 mice * ($p < 0.05$), ** ($p < 0.001$), *** ($p < 0.0001$). Haemoglobin (HGB), hematocrit (HCT), mean corpuscular volume (MCV), hemoglobin (MCH) and hemoglobin concentration (MCHC), red cell distribution width (RDW), platelets (PLT), mean platelet volume (MPV), platelet distribution width (PDW) and platelet crit (PCT)

Conclusion

Our results indicate that the normal range provided for mice by the CVM-Procell analyzer can be used for adult male and female C57BL/6J, CD1, and SCID-Beige mice.
